# Factors influencing the competence of student midwives in symphysis-fundal height measurement: A quantitative study

**DOI:** 10.4102/curationis.v49i1.2855

**Published:** 2026-04-10

**Authors:** Winile D. Cele, Pretty N. Mbeje, Euphemia M. Mhlongo

**Affiliations:** 1 Discipline of Nursing, School of Health Sciences, University of KwaZulu-Natal, Durban, South Africa

**Keywords:** competence, confidence, student midwives, symphysis-fundal height, measurement

## Abstract

**Background:**

Given the central role of antenatal competencies in midwifery practice and the critical importance of accurate symphysis-fundal height (SFH) measurement for monitoring foetal growth, it is essential to explore the factors that influence student midwives’ competence and confidence in performing this key clinical skill. Evidence showed inconsistency in SFH measurement among the midwives.

**Objectives:**

This study aimed to investigate factors influencing student midwives’ competence and confidence in the measurement, plotting and interpretation of SFH in selected public health institutions in KwaZulu-Natal.

**Method:**

A quantitative, descriptive cross-sectional design was employed. A convenience sampling technique was used to select participants. Data were collected from 184 student midwives of 250 student midwives using a structured questionnaire, yielding a response rate of 73.6%.

**Results:**

The study identified several factors influencing competence and confidence in SFH measurement, plotting and interpretation, including regular communication (74.4%), constructive feedback from clinical lecturers (76.1%), student–preceptor relationships (64.9%), role modelling (78.2%) and the overall learning environment (69.2%).

**Conclusion:**

Although many students reported receiving constructive feedback and positive role modelling, variability across units underscored the need for a more structured and standardised approach to clinical teaching and mentorship. Strengthening the clinical learning environment is essential to complement existing teaching strategies and ensure consistent, high-quality preparation of midwives.

**Contribution:**

The findings will guide curriculum developers to consider SFH skills as stand-alone procedures, so students can practise them and become competent at the point of registration as a midwife.

## Introduction

Midwifery education equips student midwives with the skills required to provide competent maternal and neonatal care. This will ensure that student midwives are competent and confident at the point of registration with the regulatory body as midwives. Symphysis-fundal height (SFH) measurement is a key antenatal competency used to monitor foetal growth (Department of Health 2017). Despite being a routine procedure required at each antenatal visit in South Africa, incomplete or inconsistent SFH recordings have been observed in maternity case records (Dlamini 2023). This gap highlighted the need to investigate the factors influencing student midwives’ competence and confidence in SFH measurement, plotting and interpretation. Consistent and accurate measurement of SFH at every antenatal visit results in early identification of risks such as intrauterine growth restriction (IUGR). If the students understand the significance of mastering SFH measurement skill, the gap identified will be bridged so that in future all SFH graphs in the maternity case records are plotted, interpreted and plan of management recorded and implemented. This will contribute to positive maternal and neonatal health outcomes. Strengthening the clinical learning environment is essential to complement existing teaching strategies and ensure consistent, high-quality preparation of midwives.

Clinical learning is a cornerstone of midwifery education, integrating theoretical knowledge with practical experience to develop professional competence. A supportive clinical environment fosters students’ confidence and skill development, preparing them for safe and accountable practice (Immonen et al. 2019). Confidence is essential in midwifery, where practitioners must make autonomous, evidence-based decisions during the antepartum, intrapartum and postpartum periods.

Studies have shown that students’ confidence and competence are influenced by effective supervision, mentoring and constructive feedback (Bäck & Karlström 2020; Sharma et al. 2018). A lack of confidence may also be associated with reduced skill performance (Bäck & Karlström 2020). Sharma et al. (2018) identified several factors influencing confidence, including student–teacher relationships, positive feedback, mentoring and student support. Their findings further indicated that most midwifery competencies and components of midwifery practice are regarded as essential. The International Confederation of Midwives (ICM 2021), in collaboration with the World Health Organization (WHO 2018), outlines four main domains of midwifery practice: antepartum, intrapartum, postpartum and newborn care. In this study, the researcher focuses specifically on the antepartum domain.

It has also been found that inconsistent support, resource constraints and negative staff attitudes can hinder clinical learning (Gemuhay et al. 2019; Mbakaya et al. 2020). Although several studies have examined students’ clinical competence, few have focused on specific technical skills, such as measuring SFH (Lawes & Jones 2020). Symphysis-fundal height measurement refers to the distance (in centimetres) between the top of the pubic bone (symphysis pubis) and the top of the uterus (fundus). It is measured using a non-elastic measuring tape placed along the pregnant woman’s abdomen. From about 20 weeks to 36 weeks of gestation, the SFH (in cm) roughly corresponds to the number of weeks of pregnancy (Papa 2022). The SFH measurement is important because it provides a simple, effective and non-invasive way to monitor the health and development of both the mother and the foetus during pregnancy. A smaller-than-expected measurement may suggest IUGR, while a larger measurement may indicate macrosomia (a large baby) or excess amniotic fluid. Helping detect abnormalities early, SFH measurement supports safe pregnancy monitoring and contributes to reducing maternal and neonatal morbidity and mortality (Pay et al. 2015). In low- and middle-income countries, SFH is often the primary or only available tool to estimate gestational age, making proficiency in this skill particularly critical (Mbakaya et al. 2020). In South Africa, government guidelines require SFH measurements at every antenatal visit to ensure proper foetal monitoring (Dlamini 2023).

Clinical competence and confidence are critical components of midwifery education, as they determine a student midwife’s ability to provide safe and effective maternal and neonatal care. Clinical learning, which combines theoretical knowledge with practical experience, plays a central role in developing essential professional skills (Immonen et al. 2019). A supportive clinical environment, along with mentoring and supervision, significantly enhances students’ confidence and competence (Immonen et al. 2019). Conversely, factors such as lack of support, poor supervision and negative attitudes from clinical staff may adversely affect skill acquisition and performance (Bäck & Karlström 2020; Sharma et al. 2018).

Several studies have examined midwifery students’ confidence across the four core domains of practice: antepartum, intrapartum, postpartum and newborn care. Research indicates that diploma students often report higher confidence levels compared to bachelor’s students, despite both groups having limited clinical exposure (Sharma et al. 2018). Key factors influencing confidence include supervision quality, relationships with midwives, positive feedback, mentoring and supportive student–teacher interactions (Almarwani & Alzahrani 2023; Bäck & Karlström 2020; Sharma et al. 2018). These findings highlight the importance of clinical support and structured learning experiences in developing self-confidence and professional competence.

Teaching methods also influence students’ confidence in performing clinical skills. Studies have shown that classroom teaching, laboratory demonstrations, supervised clinical practice and innovative approaches such as video podcasts can enhance skill acquisition and self-confidence (Sharma et al. 2018; Stone, Cooke & Mitchell 2020). Students reported high confidence in laboratory-based skills and supervised clinical placements, and preferred combining face-to-face teaching with other instructional methods to deepen understanding.

Although previous research has explored general clinical competence and confidence, few studies have focused on specific antenatal skills such as the SFH measurement. Competence in SFH measurement is essential for monitoring foetal growth, estimating gestational age and detecting potential complications during pregnancy (Department of Health 2017; Mongelli 2018; Whelan et al. 2022).

Competence in clinical practice is influenced by multiple factors, including effective supervision, structured orientation, access to resources, professional support and prior experience (Gemuhay et al. 2019; Hailu et al. 2021). Studies in Ethiopia and Tanzania have shown that students who receive orientation, staff encouragement and preceptor support demonstrate higher clinical competence and confidence, whereas those experiencing poor supervision, limited resources or absenteeism show lower performance (Gemuhay et al. 2019; Hailu et al. 2021). These findings underscore the importance of both environmental and instructional factors in shaping students’ ability to perform essential midwifery skills such as SFH measurement.

According to the South African *Nursing Act No. 33 of 2005*, a midwife must meet prescribed educational requirements, maintain competence and be registered to practice professionally. Midwives are expected to take full responsibility for maternal and neonatal health, providing care during antepartum, intrapartum periods and postpartum periods while practising ethically and within legal parameters as guided by the scope of practice, Regulation 2127 (*Nursing Act 2005*). The ICM similarly emphasises that midwives must acquire knowledge, skills and professional behaviours during training, including competencies in antenatal assessment, foetal monitoring and maternal care (ICM 2018).

### Research aim

To investigate factors influencing student midwives’ competence and confidence in the measurement, plotting and interpretation of SFH in KwaZulu-Natal of Nursing and selected public hospitals in KwaZulu-Natal.

This research forms part of a larger mixed-methods study aimed at developing guidelines for SFH measurement for student midwives. A scoping review of the literature was conducted to map the evidence on methods used in Africa for estimating gestational age during pregnancy.

## Research methods and design

### Research paradigm and design

This study was guided by pragmatism. Pragmatism is defined by Creswell and Creswell (2018) and Terre Blanche et al. (2018) as a worldview encompassing a set of philosophical assumptions that inform and guide one’s approach to inquiry. This study adopted a mixed methods design to gain an in-depth understanding of student midwives’ competence and confidence in measuring, plotting and interpreting SFH. Pragmatism allows the study to commence with a quantitative phase to generalise findings, followed by a qualitative phase to explore deeper insights (Creswell & Creswell 2018).

### Study design

The study adopted a quantitative approach, employing a descriptive cross-sectional design to investigate the factors influencing student midwives’ competence and confidence in SFH measurement, plotting and interpretation. Data were collected using Online Appendix 1 comprising closed-ended questions. Some items were adapted from previously developed instruments, including the National Competence Questionnaire (National League for Nurses 2006) and the study by Foolchand and Maritz (2020).

### Study setting

The study was conducted at five selected campuses of the KwaZulu-Natal College of Nursing (KZNCN) in KwaZulu-Natal province, South Africa. KwaZulu-Natal College of Nursing is a public nursing college accredited by the South African Nursing Council (SANC) and the Council on Higher Education (CHE), and it is registered with the South African Qualifications Authority (SAQA). The college has eleven campuses offering a range of nursing programmes. During the data collection period, the undergraduate programmes available included a four-year Diploma in Nursing (General, Psychiatric and Community) and Midwifery (SANC Regulation R425) and a three-year Diploma in Nursing (SANC Regulation R171). R171 does not include Midwifery. The five campuses that had midwifery students at the time of data collection were included in the study.

The study was also conducted in seven public hospitals across the five health districts in the province where the community service nurses included in the study were employed. Data were collected towards the end of the semester, from May 2023 to July 2023, after student midwives had completed all their theoretical modules and their comprehensive clinical examinations. At this point, the community service nurses were approximately six months post-completion. Although they are not student midwives anymore, they could provide data about their experience as student midwives. These hospitals were selected based on accessibility and cost-effectiveness. Some of the hospitals where community service nurses were allocated also served as the parent hospitals of the campuses that had students during the data collection period. A parent hospital is a hospital that provides a mandatory clinical training to affiliated nursing education institutions.

### Population

The target population comprised all student midwives enrolled in the undergraduate R425 programme who had passed the comprehensive midwifery clinical examination. In addition, community service nurses allocated to the selected public health facilities were included. Community service nurses with less than six months of experience were eligible because their level of practice was comparable to that of student midwives who had recently passed the midwifery clinical examination.

### Sample and sample size

An estimated 250 potential participants, comprising current student midwives and community service nurses, were eligible for inclusion in the study as they had already passed the clinical examination. A non-probability convenience sampling technique was employed, as participants were selected based on their availability and willingness to participate. In total, 184 participants (81 students and 103 community service nurses) provided informed consent to take part in the study.

### Data collection

After obtaining ethical clearance and institutional approval, arrangements were made with campus principals and nursing service managers to schedule suitable data-collection dates. Data were collected between May 2023 and July 2023 using a self-administered questionnaire. Participants completed the questionnaire in a quiet environment during their free time, such as during tea and lunch breaks. An information sheet was provided to ensure participants understood the study’s purpose and procedures. Those who agreed to participate in the study signed informed consent forms before data collection.

The researcher personally distributed the questionnaires and remained available throughout the process to address any questions and ensure a high response rate. Confidentiality and anonymity were maintained at all times. Completed questionnaires were securely stored under lock and key to protect participants’ information.

### Data analysis

The questionnaires were numbered and coded prior to data entry. Information was captured in an Excel spreadsheet and checked for accuracy before analysis. Demographic variables were verified, and missing data were identified and recorded. Data analysis was conducted using IBM SPSS Statistics (version 28) with assistance from a statistician. Simple descriptive univariate analysis was performed to analyse the data, and results were presented as frequencies and percentages for each variable. No statistical tests were performed because the aim was not to determine the relationship between the variables.

### Rigour

To assess the questionnaire’s reliability, Cronbach’s alpha was used to evaluate its internal consistency. The Cronbach’s alpha coefficient for the total scale was 0.94, indicating excellent reliability. Values range from zero to one, with higher values (generally above 0.7) considered acceptable (Creswell & Creswell 2018).

To establish validity of the questionnaire, the researcher sought the opinions of educational experts, the research supervisor and a statistician. In addition, an extensive review of the literature on competence and self-confidence in midwifery skills was undertaken to ensure that the instrument adequately measured the intended constructs.

### Ethical considerations

Ethical clearance was granted by the University of KwaZulu-Natal (UKZN) Biomedical Research Ethics Committee (BREC/00004960/2022) and the Provincial Department of Health (DOH) Ethics Committee (KZ_202302_027). After obtaining the ethical clearance, gatekeeper permission was also obtained from KwaZulu-Natal College of Nursing (KZNCN) and the Director, Nursing Services. Information sheets were provided to participants to ensure they understood the research process and the research ethics principles. Participants provided written informed consent before completing the questionnaire. Confidentiality was maintained during data collection, analysis and reporting of the findings.

## Results

The study’s findings are presented according to the key sections of the research instrument. Descriptive statistics, including frequencies and percentages, are used to summarise participants’ responses. Where appropriate, data are illustrated using tables, column charts and bar charts to facilitate clear visualisation of trends and patterns.

### Section A of the questionnaire

This section comprised demographic data of participants.

#### Participants’ demographics

This section presents the demographic characteristics of the participants, including gender, race, age and status (current student or community service nurse). The majority of participants were female (78.3%, *n* = 144), while males comprised 21.7% (*n* = 40). Most were black participants (82%, *n* = 151), followed by Indian participants (10.9%, *n* = 20), coloured participants (3.8%, *n* = 7) and white participants (3.3%, *n* = 6).

In terms of age distribution, 38 participants (20.7%) were between 21 years and 24 years old, while 146 participants (79.3%) were 25 years old or older. Regarding status, 44% (*n* = 81) were current students who had passed the midwifery clinical examination, and 56% (*n* = 103) were community service nurses. The detailed findings are presented in Table 1.

**TABLE 1:** Demographic characteristics of participants (*N* = 184).

Characteristics	Category	Frequency (*n*)	%
Gender	Female	144	78.3
	Male	40	21.7
Race	Black people	151	82.0
	Indian people	20	10.9
	Coloured people	7	3.8
	White people	6	3.3
Age (years)	21–24	38	20.6
	≥ 25	146	79.4
Status	Current students	81	44.0
	Community service nurses	103	56.0

### Section B of the questionnaire: Responses to symphysis-fundal height measurement, plotting and interpretation

The research instrument included several sections designed to assess different aspects of the participants’ learning experiences. These sections covered exposure to SFH measurement, unit organisation, factors influencing competence, perceptions of simulation, the lecturer’s role in clinical teaching and confidence in SFH measurement, plotting and interpretation.

Descriptive statistics, including frequencies and percentages, are presented in Online Appendix 2, and data are further illustrated using column and bar charts for easier interpretation of trends.

#### Learning experience: Symphysis-fundal height measurement

A significant majority of respondents reported that the teaching methods were helpful, with 57.1% (*n* = 105) agreeing and 32.1% (*n* = 59) strongly agreeing. This finding indicates that the instructional strategies were generally well-received and considered effective. Similarly, participants perceived simulation as beneficial in reinforcing their understanding of SFH landmarks, with 53.8% (*n* = 99) agreeing and 34.2% (*n* = 63) strongly agreeing. These findings suggest a positive impact of simulation on learning and skill acquisition.

A total of 94 respondents agreed that lectures and demonstrations were effective teaching methods, with 59 strongly agreeing. Regarding the simulation, 50.5% (*n* = 93) of respondents agreed that it covered all learning objectives, and 30.4% (*n* = 56) strongly agreed, indicating strong alignment between the simulation content and intended learning outcomes. Following the SFH measurement simulation, 51.6% (*n* = 95) agreed and 35.9% (*n* = 66) strongly agreed that they felt confident in performing SFH measurements, reflecting the simulation’s practical applicability and effectiveness in skill development.

**FIGURE 1:**
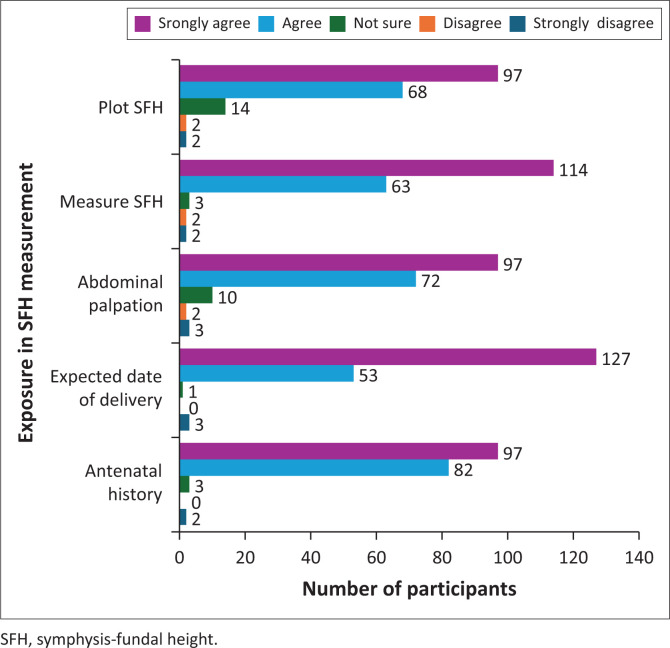
Exposure in symphysis-fundal height measurement and plotting (Part A) (*N* = 184).

**FIGURE 2:**
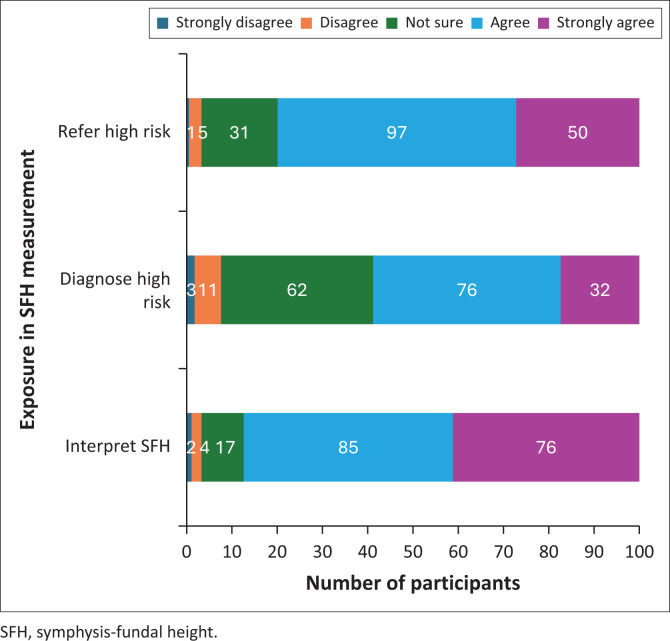
Exposure in symphysis-fundal height measurement and plotting (Part B) (*N* = 184).

As shown in Figure 1 and Figure 2, respondents reported high levels of confidence in obtaining antenatal history and performing physical examinations, with over 50% (*n* = 97) strongly agreeing that they were competent. Similarly, strong confidence was reported in calculating expected delivery dates, performing abdominal palpation, measuring SFH, and plotting findings on charts, with strong agreement ranging from 31% to 69%. Regarding confidence in interpreting SFH graphs, 41.3% (*n* = 76) strongly agreed that they felt confident. However, responses were more varied when it came to diagnosing conditions such as multiple pregnancy, polyhydramnios and IUGR, with only 17.4% (*n* = 32) strongly agreeing that they could accurately identify these conditions.

#### Unit organisation

**FIGURE 3:**
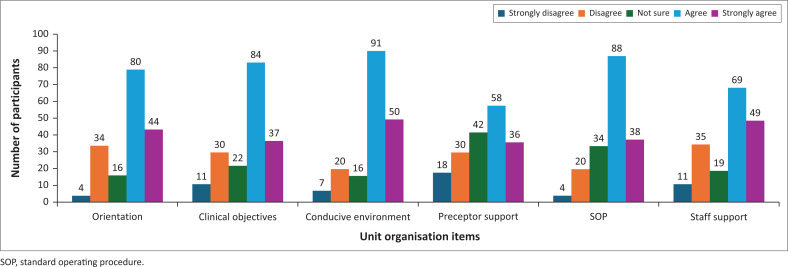
Unit organisation (*N* = 184).

As displayed in Figure 3, a large majority of respondents (67.4%) reported positive perceptions of unit organisation. Specifically, 43.5% (*n* = 80) agreed, and 23.9% (*n* = 44) strongly agreed that the orientation provided by the operational manager was satisfactory. Additionally, 49.4% (*n* = 91) agreed, and 33.6% (*n* = 60) strongly agreed that the learning environment was conducive to clinical learning.

However, some participants expressed dissatisfaction with the clarity of clinical objectives, as only 20.1% (*n* = 37) strongly agreed that objectives were clearly displayed in the unit. Regarding preceptor presence and the influence of standard operating procedures on learning, 31.5% (*n* = 58) agreed, and 19.6% (*n* = 36) strongly agreed that preceptors were available. In comparison, 32.8% (*n* = 88) agreed, and 20.7% (*n* = 38) strongly agreed that standard operating procedures positively impacted learning. Support from unit staff was rated moderately well, with 37.5% (*n* = 69) agreeing and 26.6% (*n* = 49) strongly agreeing.

#### Factors affecting competence in symphysis-fundal height measurement

**FIGURE 4:**
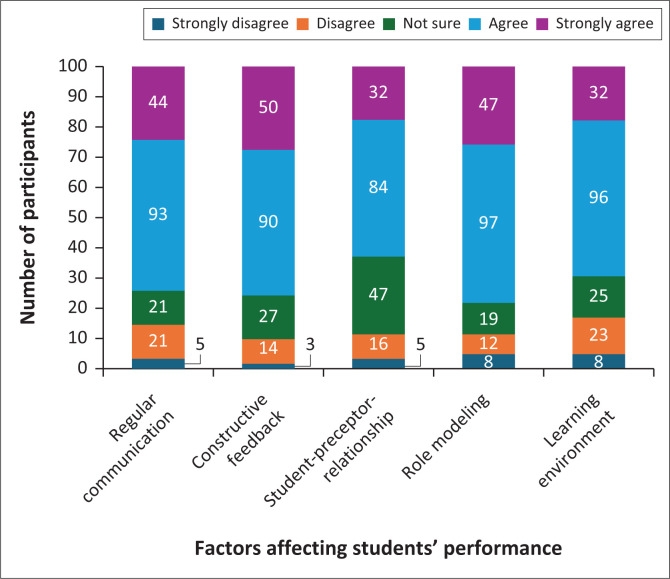
Factors affecting student midwives’ performance (*N* = 184).

Factors affecting competence are displayed in Figure 4. Regular communication and constructive feedback from clinical lecturers were perceived positively, with 50.5% (*n* = 93) agreeing and 23.9% (*n* = 44) strongly agreeing that communication was effective. Regarding constructive feedback specifically, 48.9 % (*n* = 90) participants agreed, and 27.2% (*n* = 50) strongly agreed on its positive impact.

Student–preceptor relationships also played an important role, with 47.5% (*n* = 84) agreeing and 17.4% (*n* = 32) strongly agreeing that relationships with preceptors were supportive. In this study, a preceptor is a clinical midwife who supervises the students during clinical practice and facilitates the correlation of theory to practice. However, 25.5% (*n* = 47) were unsure or disagreed, indicating potential areas for improvement.

Role modelling and the learning environment were additional factors affecting competence. Midwives were generally regarded as good role models, with 52.7% (*n* = 97) agreeing and 25.5% (*n* = 47) strongly agreeing. Regarding the learning environment maintained by clinical lecturers, 52.2% (*n* = 96) agreed, and 17.4% (*n* = 32) strongly agreed that it was conducive to learning.

#### Student views on simulation

Students generally perceived simulation activities as effective in enhancing their understanding of clinical procedures. A total of 57.6% (*n* = 106) agreed, and 30.5% (*n* = 56) strongly agreed that simulations promoted their understanding of procedures.

A high proportion of students also expressed happiness about the simulation teaching methods, with 58.7% (*n* = 108) agreeing and 23.4% (*n* = 43) strongly agreeing. In addition, the teaching materials provided were regarded as helpful, with 61.4% (*n* = 113) agreeing and only 7% (*n* = 13) disagreeing. These findings indicate that the simulations were well received and considered valuable in supporting student learning.

#### Lecturers’ role in clinical teaching

**FIGURE 5:**
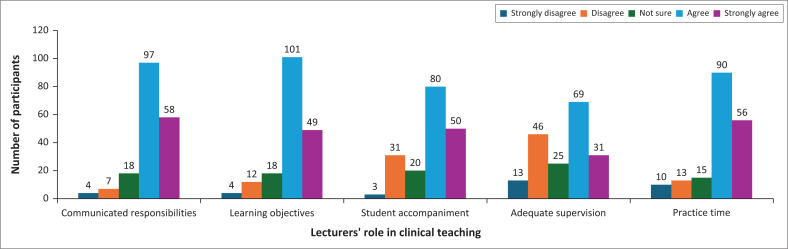
Lecturer’s role in clinical teaching (*N* = 184).

Figure 5 shows that most respondents reported that lecturers clearly communicated student midwives’ responsibilities, with 52.7% (*n* = 97) agreeing and 31.5% (*n* = 58) strongly agreeing. A small proportion disagreed (3.8%, *n* = 7) or strongly disagreed (2.2%, *n* = 4).

Lecturers were perceived to ensure that students’ work aligned with learning objectives and individual needs, with 54.9% (*n* = 101) agreeing and 26.6% (*n* = 49) strongly agreeing. Only a few respondents disagreed (6.5%, *n* = 12) or strongly disagreed (2.2%, *n* = 4).

Nearly three-quarters of respondents indicated that lecturers frequently accompanied students during clinical practice (43.5%, *n* = 80 agreeing; 27.2%, *n* = 50 strongly agreeing), though 18.4% (*n* = 34) disagreed. Regarding time spent with students and patients, only 37.5% (*n* = 69) agreed, and 16.8% (*n* = 31) strongly agreed that lecturers dedicated adequate time. In comparison, 25% (*n* = 46) disagreed, and 7.1% (*n* = 13) strongly disagreed, indicating some concerns about time allocation. A substantial majority felt that lecturers provided ample simulation practice before students engaged with real clients, with 48.9% (*n* = 90) agreeing and 30.4% (*n* = 56) strongly agreeing. Only 7.1% (*n* = 13) disagreed, and 5.4% (*n* = 10) strongly disagreed.

#### Confidence in symphysis-fundal height measurement, plotting and interpretation

**FIGURE 6:**
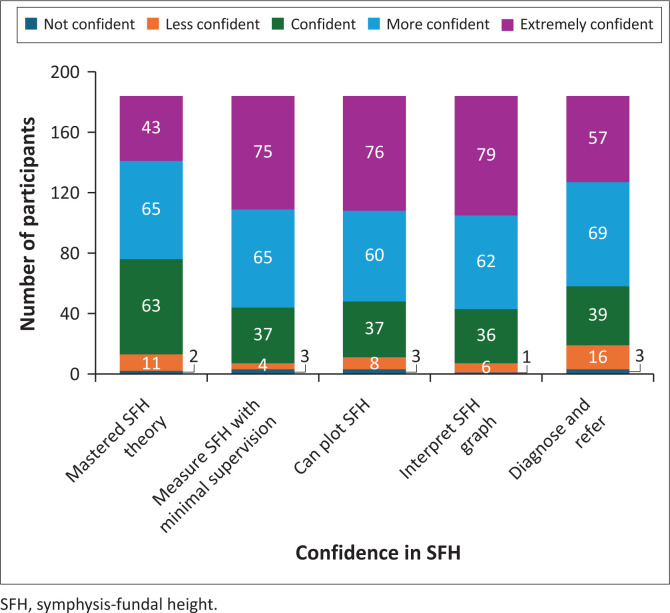
Confidence in symphysis-fundal height measurement, plotting and interpretation (*N* = 184).

Most respondents reported feeling confident in their mastery of the theoretical and clinical aspects of SFH, this is shown in Figure 6. Specifically, 34.2% (*n* = 63) were ‘confident’, 35.3% (*n* = 65) were ‘more confident’, and 23.4% (*n* = 43) were ‘extremely confident’. Collectively, over 92% of participants felt at least comfortable with the material. Only a small fraction, 1.1% (*n* = 2), reported being ‘not confident’, indicating that very few students struggled with the concepts or skills.

Findings of this study indicate that respondents’ confidence in mastering SFH theory is moderate, with 65 participants reporting being ‘more confident’ and 43 ‘extremely confident’. Confidence in measuring SFH was very high, with 35.3% (*n* = 65) ‘more confident’ and 40.8% (*n* = 75) ‘extremely confident’, suggesting strong comfort in performing SFH measurements independently. Only a small percentage, 3.8% (*n* = 7), reported feeling less confident or not confident.

Regarding plotting SFH on the SFH chart, 37 respondents felt confident to do so accurately with minimal supervision. Additionally, 32.6% (*n* = 60) were ‘more confident’, and 41.3% (*n* = 76) ‘extremely confident’, while a minority of 5.9% (*n* = 11) felt less or not confident, highlighting generally strong competence in this skill.

Overall confidence among respondents was high, with 33.7% (*n* = 62) ‘more confident’ and 42.9% (*n* = 79) ‘extremely confident’, totalling 76.6% (*n* = 141). Only 3.8% (*n* = 7) felt less or not confident in their ability to interpret the SFH graph.

Most respondents also reported confidence in making a nursing diagnosis: 21.2% (*n* = 39) felt confident, 37.5% (*n* = 69) ‘more confident’, and 31.0% (*n* = 57) ‘extremely confident’. A smaller portion, 8.7% (*n* = 16), felt less confident, suggesting that some participants may benefit from additional support in this area.

## Discussion

### Demographic data: Section A of the questionnaire

Demographic data were collected to determine the frequency distribution and percentages of the participants’ characteristics. The sample included a higher proportion of black participants compared to other racial groups, reflecting the racial distribution in the province of KwaZulu-Natal. The age of participants aligns with the South African Government bursary guidelines and learnership requirements. Females predominated in the sample, consistent with nursing being a female-dominated profession.

### Summary of the findings: Section B of the questionnaire

Midwifery education involves comprehensive clinical training, which is essential for developing student midwives’ competence and confidence. This study aimed to investigate factors influencing student midwives’ competence and confidence in measuring SFH.

In this study (Phase 2, Stage 1 of the main study), Online Appendix 3 summarised the findings related to students’ learning experiences, exposure to SFH measurement and plotting, unit organisation, lecturers’ roles and confidence levels. The factors influencing competence and confidence are discussed in detail to address the research objectives. These findings will also inform the qualitative questions in Phase 2, Stage 2 of the main study.

Simulations were particularly valued by student midwives for reinforcing learning and providing practical experience. This aligns with Vermeulen et al., 2017, cited in Folkvord and Risa (2023), who reported that simulation training can increase students’ preparedness for clinical placements, reduce anxiety and promote self-efficacy.

Lecturers were able to align learning objectives with simulation content, consistent with South African Nursing Council (SANC) requirements. Midwifery students are required to complete stipulated clinical hours and achieve specific learning objectives during placements (*Nursing Act 2005*).

Participants generally felt that the orientation provided by the operational manager was good, although some expressed dissatisfaction with the clarity of clinical objectives. Responses were mixed regarding the presence of preceptors and the influence of standard operating procedures on learning. Most respondents indicated that clinical teaching and unit organisation were beneficial, with approximately 130 participants reporting that lecturers frequently accompanied students, as required by the SANC.

Student midwives reported positive perceptions of lecturers’ roles in setting expectations, aligning learning objectives and facilitating simulations. However, there was slightly lower satisfaction regarding the frequency of lecturers’ direct involvement in clinical work, suggesting room for improvement. This is consistent with Bäck and Karlström (2020), who emphasised that student supervision is crucial, noting that stress and disengagement from supervisors can decrease confidence. Similarly, Suliman et al. (2023) highlighted that a good supervisory relationship positively influences learning.

The findings also revealed that student midwives felt more confident in certain skills, such as plotting SFH, and less confident in others, such as interpreting SFH and making nursing diagnoses. This concurs with the study by Tallam, Kaura and Mash (2021), who found that midwives were less confident in certain antenatal skills.

### Factors affecting student midwives’ competence in symphysis-fundal height measurement

To address the research objective of this study, the following factors were identified as influencing the competence of student midwives in clinical practice: communication, feedback, student-preceptor relationships, role modelling and the learning environment.

### Regular communication

Ninety-three students agreed that clinical lecturers communicated regularly with them; however, 21 disagreed, and 47 were not sure. These findings suggest that communication was effective for most students but not consistent across all clinical facilities.

Good communication skills are essential for all healthcare workers. In nursing, communication underpins the nurse–patient relationship it can positively influence health outcome (Ottonello et al. 2023). Effective communication among team members can also affect interpersonal relationship (Norman 2024). This aligns with Afriyie (2020), who defined effective communication as mutual understanding between healthcare providers and patients, promoting satisfaction for both parties. In this study this refers to communication between the lecturers and students. Grant et al. (2024) further emphasised that effective communication supports high-quality physical and holistic care. Hence, effective communication could promote high quality midwifery care by the students. Nevertheless, communication can be hindered by various barriers within clinical settings.

### Constructive feedback

Lecturers’ constructive feedback was perceived positively. Ninety students agreed, and 50 strongly agreed that they received constructive feedback. Feedback enabled students to identify areas for improvement and develop their skills. Folkvord and Risa (2023) highlighted that lecturers can support preceptors in providing effective feedback. Noble et al. (2020) noted that feedback is often given sporadically, which may affect students’ learning experiences. Constructive feedback also enhances self-esteem and confidence. Therefore, Folkvord and Risa (2023) recommended that preceptors utilise feedback skills consistently to maximise learning opportunities. In addition, constructive feedback promotes professional development and enhances quality patient care (Butler 2024)

### Student–preceptor relationships

Nearly two-thirds of students perceived their relationship with preceptors as positive; however, 84 students felt less supported. A strong student–preceptor relationship fosters a sense of belonging within the clinical team (Folkvord & Risa 2023). Preceptors can encourage students while maintaining their supervisory role. Dewar et al. (2020) reported that successful preceptor–student relationships contribute to a productive learning environment. Neiterman et al. (2022) confirmed that positive student–preceptor relationship boosted students’ confidence in clinical learning.

However, in this study some students felt preceptor support was variable. This indicates that while preceptorship was strong in many cases, there were inconsistencies across different units.

### Role modelling

Clinical midwives were generally perceived as good role models, with 97 students agreeing, and only 10.8% disagreeing. Effective role models should demonstrate competence in both cognitive and psychomotor skills. Mellish, Oosthuizen and Paton (2010) emphasised that clinical staff, unit managers and lecturers are expected to be good role models, foster team collaboration and demonstrate professionalism. This concurs with (Kurt, Turhal & Batmaz 2024) who reported that students took lecturers and clinical nurses as their role models.

Conflict among senior midwives was identified as a challenge affecting supervision (Folkvord & Risa 2023). Conversely, positive attitudes among midwives enhance student learning (Panda et al. 2021). Regular supervision by clinical instructors (clinical lecturers) supports positive learning experiences, although some students reported a lack of confidence among clinical instructors. Addressing these challenges could strengthen students’ confidence and ensure more equitable learning experiences across clinical settings.

### Learning environment

Lecturers maintained a generally positive learning environment, with 141 students reporting that it was conducive to learning. Bäck and Karlström (2020) noted that a conducive environment is essential for learning, regardless of activity. Both human and material resources contributed to the positive learning environment, enabling students to acquire clinical competence through effective teaching and simulations. In addition, workplace environment and clinical training can affect clinical competence (Almarwani & Alzahrani 2023). However, barriers existed, such as unclear display of clinical objectives in units.

Suliman et al. (2023) found that undergraduate students achieved the highest competence in value-based nursing care, while competence in leadership, and organisation of nursing was lower. This aligns with the current study’s findings, suggesting that a conducive learning environment supports the development of competence in key midwifery skills, particularly SFH measurement which is an outcome-based nursing care. According to Zhu, Jiao and Chen (2025), the nursing students’ caring behaviours and psychological resilience are promoted by the positive clinical environmental factors. This necessitate the creation of a good clinical learning environment (Zhu et al. 2025). In support of Zhu et al. (2025), improved clinical learning experiences and effective role modelling may advance the students’ caring behaviours (Inocian et al. 2022)

### Limitations

This study was conducted at the specific hospitals and specific nursing education institutions in one province; therefore, the results cannot be generalised to other provinces. A quantitative design was adopted, providing only subjective findings from participants. The study focused on factors affecting student midwives’ competence in SFH measurement, plotting and interpretation. Data were collected using questionnaires (subjective method), and no objective competence assessment was conducted due to the geographic location of the research sites and time constraints. Future research is recommended to adopt objective methods to assess competence in SFH measurement skill.

## Conclusion

This study identified factors influencing student midwives’ competence in SFH measurement, plotting and interpretation. Challenges were most pronounced in the clinical setting. There were inconsistencies, the students showed dissatisfaction with the clarity of clinical objectives which could contribute to inconsistencies in unit organisation, orientation and preceptor support limited the optimal transfer of learning into practice. While many students reported receiving constructive feedback and observing positive role modelling, variability across units highlighted the need for a more structured and standardised approach to clinical teaching and mentorship. Overall, the findings emphasise that strengthening the clinical learning environment is essential to complement existing teaching strategies and ensure consistent, high-quality preparation of midwives.
